# Snail和Claudin-3在非小细胞肺癌中的表达及意义

**DOI:** 10.3779/j.issn.1009-3419.2012.10.04

**Published:** 2012-10-20

**Authors:** 娟 李, 媛 涂, 莉莉 蒋, 缓 徐, 尚福 张

**Affiliations:** 610041 成都，四川大学华西医院病理科 Department of Pathology, West China Hospital, Sichuan University, Chengdu 610041, China

**Keywords:** 肺肿瘤, Snail, Claudin-3, 免疫组化, 组织芯片, Lung neoplasms, Snail, Claudin-3, Immunohistochemistry, Tissue microarray

## Abstract

**背景与目的:**

上皮-间质转化（epithelial mesenchymal transition, EMT）是肿瘤浸润和转移的关键步骤，上皮细胞极性丧失是其主要标志，表现为Claudin等上皮标记丢失。锌指转录因子Snail是调控EMT的重要转录因子，近年来对肿瘤侵袭转移机制研究发现Snail能提高多种肿瘤的侵袭能力。本研究旨在利用组织芯片技术探讨转录因子Snail和紧密连接蛋白（Claudin-3）在非小细胞肺癌（non-small cell lung cancer, NSCLC）及其淋巴结转移灶中的表达和意义。

**方法:**

分别采用免疫组织化学MaxVision法和EnVision法检测59例癌旁正常肺组织、302例NSCLC原发灶以及57例淋巴结转移灶中Snail和Claudin-3的表达。

**结果:**

Snail在癌旁正常肺组织、NSCLC原发灶以及淋巴结转移灶中的表达逐渐增强，差异具有统计学意义（*P* < 0.05）；Claudin-3在癌旁正常肺组织、NSCLC原发灶以及淋巴结转移灶中的表达逐渐减弱，差异具有统计学意义（*P* < 0.05）。在NSCLC原发灶中，Snail和Claudin-3的表达与肿瘤组织学类型有关（*P* < 0.05）。*Spearman*等级相关分析显示Snail与Claudin-3的表达呈负相关（*r*=-0.178, *P*=0.002)。*Kaplan-Meier*生存分析显示肿瘤大小、组织学类型、病理分级、有无癌转移、TNM分期、Snail的表达以及Snail与Claudin-3的差异性表达影响NSCLC患者的术后生存时间（*P* < 0.05）。*Cox*回归分析提示肿瘤大小、组织学类型、病理分级、有无癌转移和TNM分期是影响NSCLC患者预后的独立危险因素（*P* < 0.05）。

**结论:**

Snail和Claudin-3在NSCLC的浸润、转移中具有重要意义，有助于对NSCLC患者预后的评价。

## Abstract (en)

**Background and objective:**

Epithelial-mesenchymal transition (EMT) is considered to be one of the major molecular mechanisms inducing tumor invasion and metastasis. The loss of epithelial cell polarity is a hallmark of the EMT process. Epithelial markers such as Claudin are lost in EMT. Snail is a major transcription factor governing EMT. Recent studies about the mechanism of tumor invasion and metastasis has proven that Snail could enhance the ability of many tumors' invasiveness. The aim of this study is to investigate the expression of Snail and Claudin-3 in non-small cell lung cancer (NSCLC) and its metastatic lymph node with tissue microarray technique, and to explore clinical significance of these two molecules expression in NSCLC.

**Methods:**

Snail and Claudin-3 proteins were detected in cases of 59 adjacent normal lung tissues, 302 cases of primary NSCLC and 57 cases of lymph node metastatic tissues by MaxVision and EnVision method of immunohistochemical staining respectively.

**Results:**

The expression of Snail in adjacent normal lung tissues, NSCLC primary foci and metastatic lymph node appeared upward tendency (*P* < 0.05), and the expression of Claudin-3 in adjacent normal lung tissues, NSCLC primary foci and metastatic lymph node appeared weakened (*P* < 0.05). Further more, expression 1evel of Snail and Claudin-3 proteins in NSCLC were related to the histological type (*P* < 0.05). The Spearman correlation analysis showed that the expression of Snail and Claudin-3 was negatively correlated (*r*=-0.178, *P*=0.002). *Kaplan-Meier* survival analysis indicated that factors such as the tumor size, histological type, grading, metastasis, TNM staging, and difference expression between two proteins were associated with the postoperative survival rate of NSCLC patients (*P* < 0.05). Multivariate analysis using *Cox* regression model identified that the tumor size, histological type, grading, metastasis and TNM staging were independent prognostic factors of NSCLC patients (*P* < 0.05).

**Conclusion:**

Snail and Claudin-3 may play important roles in invasion and metastasis in NSCLC, So might be employed to evaluate the prognosis of NSCLC patients.

肺癌是最常见的恶性肿瘤之一。全世界每年有超过一百万人死于肺癌，占癌症总死亡人数的26%-29%^[1]^。其中，80%的患者为非小细胞肺癌（non-small cell lung cancer, NSCLC）。由于早期症状不典型，多数患者就诊时已处于晚期，导致其预后差，5年生存期约为15.6%^[2]^。上皮间质转化（epithelial mesenchymal transition, EMT）被认为是肿瘤浸润和转移的关键步骤，锌指转录因子Snail是调控EMT的重要转录因子。它主要通过与上皮钙粘素（E-cadherin）启动子区的E-box连接基序结合，抑制E-cadherin转录表达，同时直接抑制紧密连接蛋白（Claudin）的表达，诱导EMT发生。近年来对肿瘤侵袭转移机制研究发现Snail能提高多种肿瘤的侵袭能力。Claudin是构成紧密连接（tight junction, TJ）的骨架蛋白，有24个异构体，具有参与维持细胞的屏障功能，决定细胞旁物质选择渗透性和细胞极化等重要生理功能，其表达数量和分布结构的变化直接影响TJ的结构和功能^[3, 4]^。Claudin-3是其家族成员。人类Claudin-3定位于7q11.23。人体内乳腺导管上皮细胞、卵巢上皮细胞、子宫内膜、食管黏膜、胰腺腺泡细胞及导管和胰岛细胞、前列腺上皮细胞、支气管和肺泡上皮细胞中均有Claudin-3的表达。近期研究发现Claudin-3在某些肿瘤，如乳腺癌、卵巢癌、前列腺癌、胃癌和食管腺癌的mRNA和蛋白表达均明显增高^[5-10]^，而在大肠癌中表达下调，其具体机制尚不完全清楚^[11]^。

本实验利用组织芯片技术和免疫组织化学染色检测Snail和Claudin-3在癌旁正常肺组织、NSCLC组织及其淋巴结转移灶中的表达情况，探讨两者在NSCLC中表达的意义，为其应用于NSCLC的诊断和预后判断提供依据。

## 1 资料与方法

### 1.1 标本来源

收集四川大学华西医院1998年1月-2003年12月手术切除的NSCLC病例，从中选出临床资料完整和石蜡组织保存完好的病例共302例，组织学类型包括鳞状细胞癌（以下简称鳞癌）、腺癌、腺鳞癌和大细胞癌。其中伴有转移灶146例，实际收集其淋巴结转移性癌组织57例。另取同期手术的59例癌旁正常肺组织作为对照。患者截至术前均未行放疗或化疗。所有标本均由病理学教授带领复习HE染色切片，明确病理诊断。302例NSCLC标本均经切片、HE染色，明确病理诊断（2003年WHO肺肿瘤组织学分类标准）。其中，男性243例，女性59例，男：女为4.12:1。发病年龄35岁-82岁，中位年龄61岁。鳞癌138例，腺癌124例，腺鳞癌33例，大细胞癌7例。其中高分化癌（包括高分化鳞癌、高分化腺癌）20例；中分化癌（包括中分化鳞癌、中分化腺癌）131例；低分化癌（包括低分化鳞癌、低分化腺癌、腺鳞癌、大细胞癌）151例。TNM分期，Ⅰ期98例（Ⅰa期10例，Ⅰb期88例），Ⅱ期71例（Ⅱa期25例，Ⅱb期46例），Ⅲ期112例（Ⅲa期92例，Ⅲb期20例），Ⅳ期21例（2009年肺癌国际分期修订版）。生存时间0.06个月-96个月，中位生存时间35.3个月。Ⅰ期患者的5年生存率为37.8%，Ⅱ期为32.9%，Ⅲ期为8.0%，Ⅳ期患者基本都在2年内死亡。生存期的计算从手术日期起到随访日期或由于复发、转移而死亡的日期为止。

### 1.2 组织芯片的制作

采用手工制作组织芯片。首先根据HE染色切片观察，确定代表性病变部位后在组织切片和相应石蜡组织块上标志，并在空白石蜡块上钻孔，组织片直径与孔径一致（内径为1.5 mm），再用金属空心管从经定位的目标石蜡块上钻取组织并转移至已钻孔的空白石蜡块的相应位置。连续切片后，将切下的5 μm厚组织裱于经防脱片处理的载玻片上，置于56 ℃烤箱中烘烤72 h。常温保存备用。

### 1.3 免疫组织化学染色

兔抗人Snail多克隆抗体和兔抗人Claudin-3多克隆抗体分别购自北京博奥森生物技术有限公司和福州迈新生物技术开发有限公司。免疫组化试剂盒和DAB显色试剂盒均购自福州迈新生物技术开发有限公司。Snail一抗的工作稀释浓度为1:50，实验采用MaxVision法；Claudin-3为工作液，实验采用EnVision法进行免疫组化染色，具体步骤严格按照产品说明书进行。分别用已知阳性的胃腺癌组织和结肠腺癌组织作为Snail和Claudin-3的阳性对照，以0.01 mol/L PBS（pH7.4）代替一抗作为阴性对照。

### 1.4 免疫组织化学结果判定

Snail和Claudin-3以细胞核或/和细胞浆出现棕黄色为阳性细胞。每张切片高倍镜下随机选取10个视野共记录1, 000个细胞，综合染色强度和阳性细胞数量进行判定。①按切片中细胞着色深浅评分：0分为细胞无显色；1分为黄色；2分为棕黄色；3分为棕褐色。②按阳性细胞数占同类细胞数的百分比评分：0分为 < 5%；1分为5%- < 25%；2分为25%- < 50%；3分为50%- < 75%；4分为≥75%。取①、②两项评分的乘积作为总积分：0分为阴性（-）；1分-4分为弱阳性（+）；5分-8分为中等强度阳性（++）；≥9分为强阳性（+++）^[12]^。

所有染色结果的判定和计数均采用统一的评分标准，在对样本临床资料完全不知的情况下，由两名研究者分别对实验结果进行判断、评分。若两人评定结果相差3分则重新评定。

### 1.5 统计分析

等级资料采用秩和检验，采用*Spearman*等级相关分析，应用*Kaplan-Meier*法（组间生存率比较采用*Log-rank*检验）和*Cox*比例风险回归模型进行生存分析。*P* < 0.05为差异有统计学意义。

## 2 结果

### 2.1 Snail和Claudin-3在癌旁正常肺组织、NSCLC原发灶和淋巴结转移灶中的表达

Snail和Claudin-3阳性信号定位于细胞质或/和细胞核。两种蛋白阳性表达均见于支气管上皮和Ⅱ型肺泡上皮细胞。在间质组织中也可见Snail和Claudin-3的阳性信号（图 1）。在NSCLC原发灶和淋巴结转移灶中表达强度不同（图 2，图 3)。

**1:**
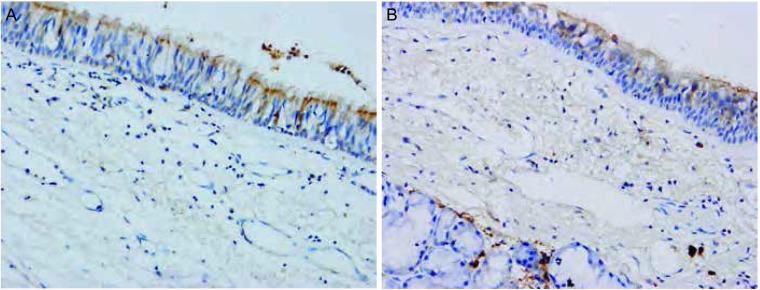
Snail和Claudin-3在癌旁正常肺组织中的表达（A: Snail, MaxVision; B: Claudin-3, EnVision; ×400） Expression of Snail and Claudin-3 in normal lung tissues (A: Snail, MaxVision; B: Claudin-3, EnVision; ×400)

**2:**
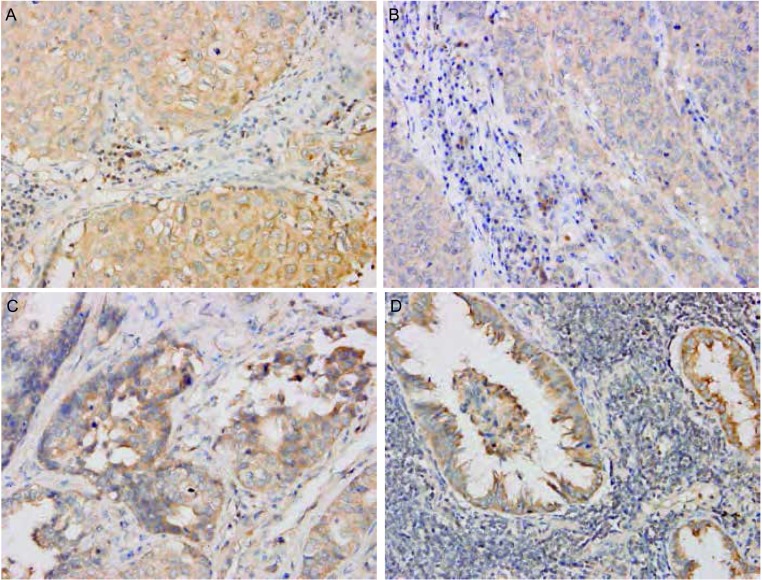
Snail在NSCLC原发灶及淋巴结转移灶中的表达（MaxVision, ×400） Expression of Snail in NSCLC primary foci and lymph node metastases (MaxVision, ×400). A: Squamous cell carcinoma; B: Lymph node metastases of squamous cell carcinoma; C: Adenocarcinoma; D: Lymph node metastases of adenocarcinoma. NSCLC: non-small cell lung cancer

**3:**
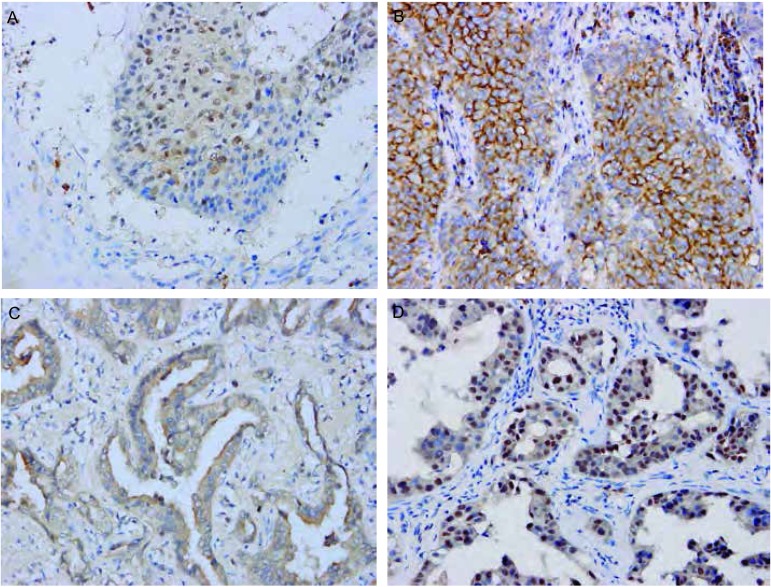
Claudin-3在NSCLC原发灶及淋巴结转移灶中的表达（EnVision, ×400） Expression of Claudin-3 in NSCLC primary foci and lymph node metastases (EnVision, ×400)

Snail在癌旁正常肺组织、NSCLC原发灶以及淋巴结转移灶中的表达逐渐增强，阳性率分别为47.5%（28/59）、92.1%（278/302）及96.5%（55/57），差异具有统计学意义（χ^2^=89.097, *P* < 0.05)。两两比较发现，Snail在NSCLC原发癌组织中的阳性表达率高于癌旁正常肺组织（*P* < 0.05）、低于淋巴结转移癌组织（*P* > 0.05）。Claudin-3在癌旁正常肺组织、NSCLC原发灶以及淋巴结转移灶中的表达逐渐减弱，阳性率分别为88.1%（52/59）、60.3%（182/302）及45.6%（26/57），差异具有统计学意义（χ^2^=18.667, *P* < 0.05）。两两比较发现，Claudin-3在NSCLC原发癌组织中的阳性表达率分别低于癌旁正常肺组织（*P* < 0.05）、高于淋巴结转移癌组织（*P* > 0.05）（表 1）。

**1:** Snail和Claudin-3在癌旁正常肺组织、NSCLC原发灶及淋巴结转移灶中的表达 Expression of Snail and Claudin-3 in normal lung tissues, NSCLC primary foci and lymph node metastases

Tissue type	*n*	Snail		*P*	Claudin-3		*P*
		-	+-+++		-	+-+++	
Normal lung tissues	59	31	28	< 0.001	7	52	< 0.001
NSCLC primary foci	302	24	278		120	182	
Lymph node metastases	57	2	55		31	26	

### 2.2 Snail和Claudin-3在NSCLC原发灶中的表达与临床病理特征之间的关系

NSCLC原发灶中，Snail和Claudin-3的表达均与肿瘤组织学类型（χ^2^=9.443, *P*=0.024; χ^2^=23.863, *P* < 0.05）有关。而与患者的年龄、性别以及肿瘤大小、病理分级、有无癌转移和TNM分期无关（表 2）。

**2:** Snail和Claudin-3的表达与NSCLC临床病理特征的关系 Relationship between expression of Snail and Claudin-3 and pathological charaeteristics of NSCLC

Characteristic	*n*	Snail		*P*	Claudin-3		*P*
		-	+-+++		-	+-+++	
Gender							
Male	243	20	223	0.712	90	153	0.129
Female	59	4	55		17	42	
Age (yr)							
≤61	156	13	143	0.798	61	95	0.798
＞61	146	11	135		55	91	
Tumor size (cm)							
≤3	113	5	108	0.080	39	74	0.282
> 3	189	19	170		77	112	
Histological type							
Squamous cell carcinoma	138	18	120	0.024	69	69	< 0.001
Adenocarcinoma	124	4	120		38	86	
Denosquamous carcinoma	33	2	31		4	29	
Large cell carcinoma	7	0	7		5	2	
Grading							
Well	20	1	19	0.104	7	13	0.937
Moderate	131	6	125		50	81	
Poor	151	17	134		59	92	
Metastasis							
Yes	146	10	136	0.495	55	91	0.798
No	156	14	142		61	95	
TNM staging							
Ⅰ+Ⅱ	169	13	156	0.854	69	100	0.330
Ⅲ+Ⅳ	133	11	122		47	86	

### 2.3 Snail和Claudin-3在NSCLC组织中表达的相关性

*Spearman*等级相关分析显示，Snail和Claudin-3在NSCLC组织中的表达呈负相关（*r*=-0.178, *P*=0.002）。

### 2.4 Snail和Claudin-3表达与预后的关系

#### 2.4.1 *Kaplan-Meier*单因素生存分析

将Snail和Claudin-3表达分为表达（+-+++）和不表达（-）两组。Snail表达组和不表达组5年生存率分别为8.3%和0.7%；Claudin-3表达组和不表达组5年生存率分别为6.0%和3.0%。采用*Kaplan-Meier*法绘制NSCLC患者生存曲线，*Log-rank*检验不同样本的生存曲线，结果显示（图 4，图 5）：Snail不同表达之NSCLC患者的生存率差异无统计学意义（χ^2^=1.539, *P*=0.215）。Claudin-3不同表达之NSCLC患者的生存率差异无统计学意义（χ^2^=2.942, *P*=0.086）。

**4:**
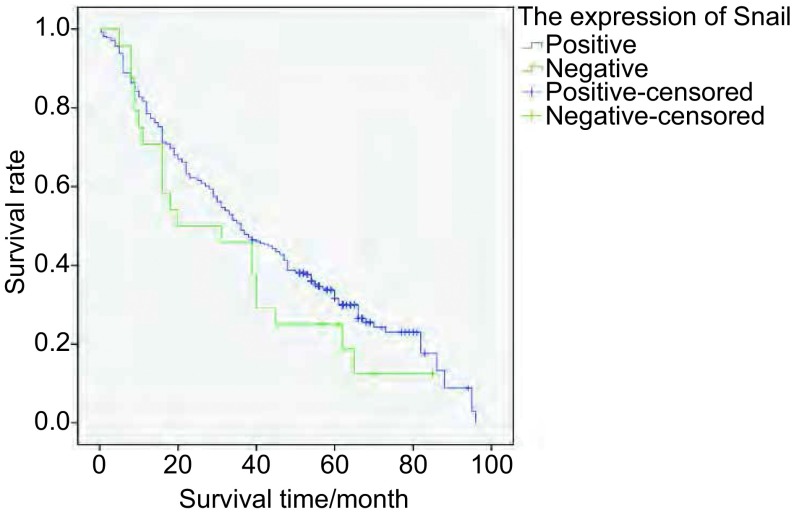
Snail表达组和Snail不表达组NSCLC患者的*Kaplan-Meier*生存曲线 *Kaplan-Meier* survival curves of NSCLC patients with or not expression of Snail

**5:**
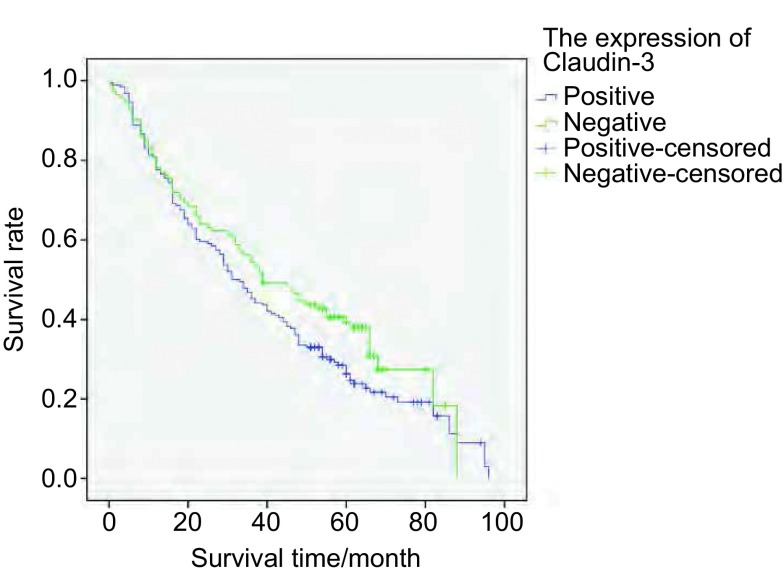
Claudin-3表达组和Claudin-3不表达组NSCLC患者的*Kaplan-Meier*生存曲线 *Kaplan-Meier* survival curves of NSCLC patients with or not expression of Claudin-3

#### 2.4.2 *Cox*比例风险回归模型分析

对患者的年龄、性别、组织学类型、分化程度、TNM分期、淋巴结转移、Snail和Claudin-3的表达等可能与预后有关的指标进行分析。采用前向逐步法，显示在α=0.05的水平上，肿瘤大小、组织学类型、病理分级、有无癌转移、TNM分期这5个因素被筛入*Cox*模型内（表 3），是影响NSCLC患者预后的独立危险因素。其中，肿瘤大小、组织学类型、有无癌转移和TNM分期的偏回归系数为正值，表明肿瘤越大、分化程度越低和伴有癌转移，患者死亡的风险越大。病理分级的偏回归系数为负值，说明分化愈低，患者死亡风险愈高。由相对危险度得知：NSCLC的肿瘤直径 > 3 cm的患者其死亡风险是直径≤3 cm患者的1.68倍；伴有癌转移的患者其死亡风险较不伴有癌转移患者增加了58.5%；随着肿瘤分级由高到低的变化，患者的死亡风险依次增加31.6%。随着TNM分期的递增，患者的死亡风险依次增加52.5%。NSCLC患者的性别（*P*=0.639）、年龄（*P*=0.783）、Snail的表达（*P*=0.561）、Claudin-3的表达（*P*=0.265）和Snail与Claudin-3的差异性表达（*P*=0.265）对患者预后的影响无统计学意义。

**3:** *Cox*回归模型筛选的影响NSCLC患者的危险因素 The risk fact of NSCLC patients selected by *Cox* regression model

	*β*	SE	Wald	*P*	Exp (*β*)
Tumor size	0.519	0.165	9.847	0.002	1.680
Histological type	0.502	0.163	9.447	0.002	1.651
Grading	-0.380	0.131	8.426	0.004	0.684
Metastasis	0.461	0.234	3.890	0.049	1.585
TNM staging	0.425	0.131	4.520	0.030	1.525

## 3 讨论

EMT是指细胞由上皮表型向间质表型转化的过程，具有转分化特征，即上皮细胞极性丧失及其间质特性获得。上皮细胞极性丧失表现为上皮标记如E-cadherin、Claudin、Occludins和Muc1的丢失，是EMT的重要特征之一。E-cadherin是上皮细胞表面的粘附分子，对于细胞之间的粘附起到重要的作用。Claudin是构成TJ的骨架蛋白，对维持细胞完整性起着至关重要的作用^[5]^。E-cadherin和Claudin表达下调常使细胞之间的粘附能力下降、凝聚力降低、分化变差、转移和侵袭能力增强。此外，Claudin-3不仅具有调节细胞间物质流动和维持上皮细胞极性的功能，还参与细胞增殖分化、基因转录、肿瘤抑制等过程。故在肿瘤发生、发展及转移过程中发挥重要作用。

研究发现，Claudin在不同的肿瘤组织中发挥不同的作用，表达也不相同。Claudin-3在乳腺癌、前列腺癌、卵巢癌、胃癌和食管癌中表达上调^[6-9]^，而在大肠癌及NSCLC中表达下调^[10, 11]^。

本实验结果显示，在59例癌旁正常肺组织中，Claudin-3的阳性表达见于支气管上皮和Ⅱ型肺泡上皮细胞。在302例NSCLC原发癌组织中，Claudin-3的阳性表达率为60.3%，Claudin-3在NSCLC组织中的阳性表达率低于癌旁正常肺组织，其表达与肿瘤的组织学类型有关，而与患者的性别、年龄、肿瘤大小、病理分级、有无转移以及TNM分期无关。Claudin-3在不同组织学类型NSCLC中的表达各不相同，具体阳性表达率如下：在鳞癌中为50%；在腺癌中为69.3%；在腺鳞癌中为87.9%；在大细胞癌中为28.6%。其表达率由高到低分别为腺鳞癌、腺癌、鳞癌和大细胞癌。其他学者的研究也证实Claudin-3在不同组织学类型肺癌中的表达存在差异，这种差异有助于肺癌组织学类型的区分^[12]^。有研究^[13]^发现Claudin-3的表达与前列腺癌的临床病理分期及复发密切相关，在Ⅲ期、Ⅳ期肿瘤组织中的表达高于Ⅰ期、Ⅱ期，在复发肿瘤中的表达高于无复发肿瘤。本实验中，Claudin-3在NSCLC原发癌组织中Ⅰ期、Ⅱ期患者中表达率低于在Ⅲ期、Ⅳ期患者中的表达率，其差异无统计学意义。

EMT的另外一个重要方面是间质特性的获得，即间质标记如成纤维细胞样的外形、波形纤维蛋白、Snail、骨桥蛋白基因的表达。Snail是锌指转录因子超家族成员，其主要功能是调控EMT。*E-cadherin*被认为是Snail直接作用的靶基因。Snail主要以锌指区域与E-cadherin启动子上的E-box序列结合，达到抑制E-cadherin转录的作用。肿瘤发生EMT时，E-cadherin的表达降低或缺失、细胞间的粘附力减弱、细胞之间彼此分离、获得活动能力并抵抗凋亡，肿瘤细胞易于从原发灶上脱落。而肿瘤细胞从原发部位脱落是肿瘤浸润和转移的第一步，也是EMT发生的标志。Snail通过抑制E-cadherin、Claudin-3等的表达来促进EMT的发生，在肿瘤的浸润转移中起重要作用。

研究发现，Snail在乳腺癌、食管鳞癌、肺癌等多种肿瘤中表达上调^[14-19]^，并与肿瘤的组织学类型、病理分级、侵袭转移、生存时间等有关。

本研究显示，59例癌旁肺组织中，Snail在支气管上皮和Ⅱ型肺泡上皮细胞呈阳性表达；在302例NSCLC中，Snail阳性表达率为92.1%。Snail在NSCLC中的表达高于正常肺组织，与文献报道一致^[18]^。Snail在302例NSCLC原发癌组织中的表达与患者的组织学类型有关，而与患者的性别、年龄、肿瘤大小、病理分级、有无转移以及TNM分期无关。Snail不同组织学类型NSCLC中表达不同，具体阳性表达率如下：在鳞癌中为87%；在腺癌中为96.8%；在腺鳞癌中为94%；在大细胞癌中为100%。其表达率由高到低分别为大细胞癌、腺癌、腺鳞癌和鳞癌。本实验结果提示，在NSCLC中，Snail可能具有促进肿瘤细胞的转移和肿瘤演进的作用，尚有待进一步深入研究证实。

本研究显示，Snail和Claudin-3在NSCLC组织中的表达呈负相关，提示两种蛋白在肿瘤的发生、发展中起不同的作用。

目前，有学者在研究肺癌靶向治疗时将目光瞄准了Snail和Claudin-3。如通过siRNA靶向干扰Snail的表达可增加顺铂诱导的A546肺癌细胞的凋亡，联合化疗药物和靶向抑制Snail因子大大提高了肺癌治疗的有效性^[20]^；或者通过siRNA沉默*Snail*基因的表达或通过反义Snail阻止EMT和肿瘤的转移，利用化疗药物抑制Snail蛋白的生成，可望能有效地抑制肿瘤的侵袭和转移，从而大大提高抗肿瘤效力。而围绕Claudin-3靶向治疗的研究主要在于两个方面：抗Claudin抗体的制备和利用肉毒素产气荚膜杆菌（clostridiumperfringens enterotoxin, CPE）定位Claudin。前者基于利用抗Claudin抗体对抗Claudin的细胞外域。后者利用CPE与Claudin-3的结合，CPE是分子量为35 kDa的单链多肽，其功能区由N-末端的细胞毒域和C-末端的受体结合域组成。Claudin-3作为CPE的受体与其结合，CPE结合在Claudin-3的第二个细胞外环。将从铜绿假单胞菌外毒素（pseudomonas aeruginosa exotoxin）中提取的蛋白合成抑制因子PSIF与CPE的C-末端（C-CPE）融合形成Claudin-3靶向分子C-CPE-PSIF。此分子能识别细胞极性，且肿瘤内注射C-CPE-PSIF能抑制肿瘤生长。所以，C-CPE可能成为药物输送和肿瘤治疗的一个新分子^[21]^。研究证实，C-CPE与肿瘤坏死因子（tumor necrosis factor, TNF）融合后可抑制卵巢癌细胞生长，CPE片段可能成为Claudin靶向治疗的工具^[22]^。实验中利用Claudin-3 siRNA可抑制卵巢癌小鼠肿瘤的生长及转移。因此，siRNA介导的*Claudin*基因沉默也是潜在的抗肿瘤药物^[23]^。Claudin靶向治疗可能使NSCLC的治疗取得突破性的进展^[24]^。

综上所述，Snail和Claudin-3的表达与NSCLC的发生、发展密切相关，可以作为判断NSCLC淋巴结转移能力的生物学指标，对肿瘤预后的判断有一定的参考价值。同时，在肿瘤的靶向治疗中具有潜在应用价值。
